# Use of electron backscatter diffraction patterns to determine the crystal lattice. Part 3. Pseudosymmetry

**DOI:** 10.1107/S1600576723000845

**Published:** 2023-02-24

**Authors:** Gert Nolze, Tomasz Tokarski, Łukasz Rychłowski

**Affiliations:** a Federal Institute for Materials Research and Testing (BAM), Unter den Eichen 87, 12205 Berlin, Germany; b Institut für Mineralogie, TU Bergakademie Freiberg, Brennhausgasse 14, 09596 Freiberg, Germany; c Academic Centre for Materials and Nanotechnology, AGH University of Science and Technology, Mickiewicza 30, 30-059 Krakow, Poland; Ecole National Supérieure des Mines, Saint-Etienne, France

**Keywords:** Bravais lattices, pseudosymmetry, lattice point density, ordered/disordered structures, lattice distortion, electron backscatter diffraction, backscattered Kikuchi diffraction patterns, lattice parameters, Funk transform

## Abstract

A pseudosymmetric description of the crystal lattice derived from a single wide-angle Kikuchi pattern can have several causes. The small size (<15%) of the sector covered by an electron backscatter diffraction pattern, the limited precision of the projection centre position and the Kikuchi band definition are crucial. Inherent pseudosymmetries of the crystal lattice and/or structure also pose a challenge in the analysis of Kikuchi patterns. To eliminate experimental errors as much as possible, simulated Kikuchi patterns of 350 phases have been analysed using the software *CALM* [Nolze *et al.* (2021). *J. Appl. Cryst.* **54**, 1012–1022] in order to estimate the frequency of and reasons for pseudosymmetric crystal lattice descriptions. Misinterpretations occur in particular when the atomic scattering factors of non-equivalent positions are too similar and reciprocal-lattice points are systematically missing. As an example, a pseudosymmetry prediction depending on the elements involved is discussed for binary *AB* compounds with *B*1 and *B*2 structure types. However, since this is impossible for more complicated phases, this approach cannot be directly applied to compounds of arbitrary composition and structure.

## Abstract 2

Distinguishing between actual and apparent pseudosymmetry in electron backscatter diffraction patterns is nearly impossible, even for simulated patterns. However, the resulting lattice is always a superlattice as long as the signal is not a superposition of multiple patterns.

## 1. Introduction

Symmetry is a fundamental concept in crystallography that combines rotations and/or rotoinversions within the crystal structure with the omnipresent translational periodicity of a crystal lattice. The point-group symmetry is a minimal symmetry requirement in every physical property and also theoretically in a captured signal like an electron diffraction pattern. Unfortunately, the ideal crystal symmetry is often overlaid by an asymmetry caused by the measurement setup, which, strictly speaking, leads to its loss. In the case of an electron backscatter diffraction (EBSD) signal, this asymmetry is the consequence of the distinct direction of the primary electron beam and the tilt of the sample, which produces, among other things like a high gnomonic distortion, significant excess-deficiency phenomena and a comparatively strong radial intensity decay in the EBSD pattern. After background correction a backscattered Kikuchi diffraction (BKD) pattern results, which may look nearly perfect at first glance. A closer look shows a radial decrease in the signal-to-noise ratio, which is of course inconsistent with the crystal symmetry of the pattern-forming phase, and which additionally varies with the projection centre (PC) position and a possibly existing microscopic surface topography.

Although the solid angle reflected from a typical wide-angle BKD pattern is considered quite large compared with electron channelling patterns, the acquired image still represents <15% of the total diffraction signal given by a simulated master pattern. This considerably reduces at least the purely visual detectability of symmetry for non-cubic phases. The incomplete signal also affects the reliability of the extracted information such as the lattice parameters, because even mirror-symmetric band *profiles* of centrosymmetric phases become highly asymmetric due to the large missing part of the bands [Part I of this series, Nolze *et al.* (2023*a* ▸)]. This also happens for physics-based BKD pattern simulation, although they are at least not affected by excess-deficiency effects and the already-mentioned gradually changing signal-to-noise ratio.

Such discrepancies from theoretically highly symmetrical signals are particularly disturbing when small symmetry deviations are to be investigated. They are referred to as pseudosymmetry, which describes any state in which the difference from another symmetry is so small that neither higher nor lower symmetry can be clearly distinguished with the measurement technique applied.

**Figure 1:**
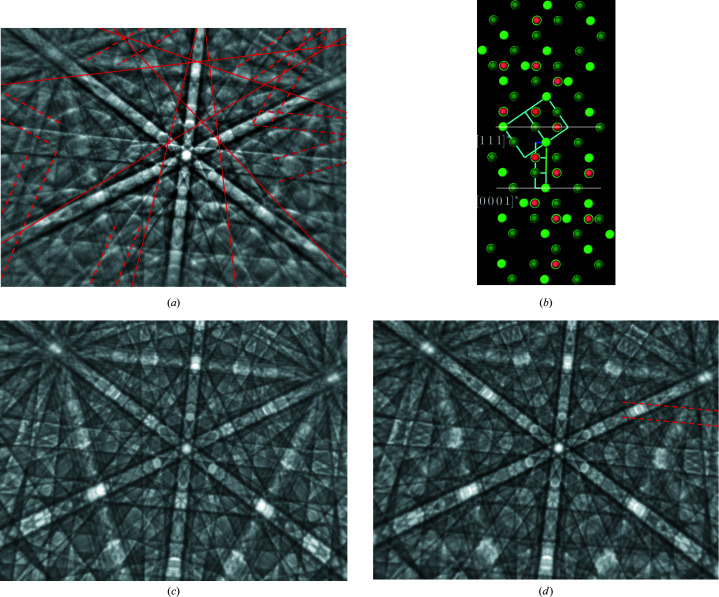
(*a*) The BKD signal collected on zincblende (ZnS) very likely twinned || {111}. The twinned part of the signal is identifiable by 14 faint but clearly visible additional bands. Their band edges are indicated by dashed red lines, and for bands invisible to the eye the lattice plane traces are plotted as solid red lines. (*b*) The marked bands in panel (*a*) are shown as red spheres and are responsible for the sketched hexagonal reciprocal cell with (0001) || (111)_c_. Without the red spheres the correct (centred) cubic cell results (projection 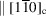 ). The reciprocal-lattice points at the corners of the cubic unit cell are highlighted in light green, while the dark-green spheres display centred positions. (*c*) The simulated pattern of zincblende and (*d*) the overlaid signal of two twinned ZnS crystals with an intensity ratio of 100:60, where one clearly visible false Kikuchi band marked by red dashed lines is missing in panel (*c*) but included in (*a*).

Therefore, in the context of the determination of lattice parameters from individual BKD patterns, the causes of pseudosymmetry may be mainly classed as one of the following:

(i) *Phase specific*. Pseudosymmetry indicates a state of apparently higher symmetry density, *i.e.* an apparent increase in the number of symmetry operators per unit-cell volume. Thus, either the lattice point density (*translationengleiche* or *t* supergroups) or the point-group symmetry of each individual lattice point (*klassengleiche* or *k* supergroups) appears higher than it actually is [see *e.g.* Hahn (2005 ▸), Section 8.3.3.3, p. 735, or Wondratschek & Müller (2004 ▸), Section 2.1.6, p. 63]. In practice, however, the two processes often interfere. For example, superstructures are the result of any kind of atomic rearrangement during diffusive, reactive or thermodynamically controlled processes that do not simply reduce the lattice point density; they are also responsible for additional, often very small, lattice distortions that may eliminate previously existing symmetry operators (Howard & Stokes, 2005 ▸; Capillas *et al.*, 2005 ▸, 2011 ▸).

(ii) *Microstructure specific*. Pseudosymmetry may also appear as a consequence of specific microstructures or structure defects, *e.g.* caused by micro- or nano-twinning, antiphase boundaries, stacking faults *etc.* (Fischer *et al.*, 2021 ▸). In such cases, perfectly aligned BKD patterns overlap (Lenthe *et al.*, 2020 ▸). Because of high lattice correlations the resulting BKD pattern may reflect a pronounced pseudosymmetry, which may effectively also result in a lower symmetry. In Fig. 1 ▸, cubic ZnS is misinterpreted as some bands do not fit the cubic lattice. However, all of them fit to a hexagonal superlattice.

(iii) *Method specific*. Whether or not a phase is considered pseudosymmetric depends on the limitations of the analysis or post-processing method used. For example, a lattice distortion which is easily identifiable by interference splitting in X-ray powder diffraction can end in a merely pseudosymmetric description by less angular resolving techniques like optical reflection goniometry or EBSD [from the different angular resolution limits it already follows that EBSD cannot produce more accurate results than X-ray diffraction (XRD)].

In the following, we will focus exclusively on the pseudosymmetry caused by the phase itself.

Typical pseudosymmetry limitations that appear during the analysis of BKD patterns are discussed *e.g.* by Zambaldi *et al.* (2009 ▸), Nolze *et al.* (2015 ▸, 2016 ▸), Schmidt *et al.* (2016 ▸), Callahan *et al.* (2018 ▸), Jackson *et al.* (2018 ▸), De Graef *et al.* (2019 ▸), Pang *et al.* (2020 ▸), Lachmann *et al.* (2020 ▸) and Martin *et al.* (2022 ▸). Despite different approaches, the goal is almost always to eliminate as much as possible the observed pseudo­symmetry of a phase in orientation maps. Depending on the strength of the pseudosymmetry, this is achieved either with very moderate means or with considerable effort. In very complicated cases, detection often involves additional assumptions, such as a PC known to be perfect, the exclusion of any distortion in the BKD pattern or the use of Kikuchi bands that are virtually invisible to the naked eye (Martin *et al.*, 2022 ▸).

Sometimes, an improper pattern interpretation setup during orientation mapping can also lead to multiple solutions of the same BKD pattern. This is also sometimes called pseudosymmetry of actually non-pseudosymmetric phases (Nowell & Wright, 2005 ▸; Vaudin, 2005 ▸; Karthikeyan *et al.*, 2013 ▸); it can, however, easily be avoided.

### 1.1. Crystallographic pseudosymmetry

#### 1.1.1. Crystal lattice metric

**Figure 2:**
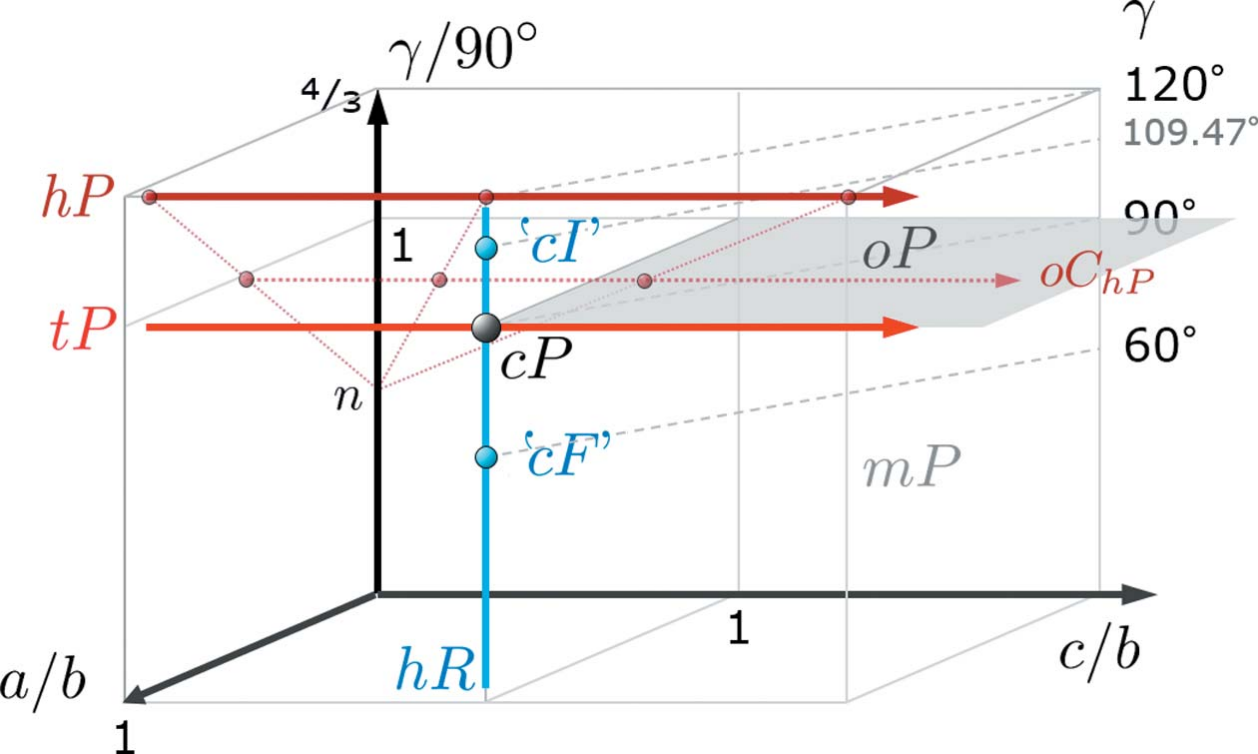
The relationships between non-triclinic primitive Bravais lattices using *a*/*b*, *c*/*b* and γ. The primitive cell descriptions of *cI* and *cF* are displayed, as well as *oC_hP_* as an alternative orthorhombic (centred) description of *hP*.

Pseudosymmetry is very often assumed to be equivalent to minimal lattice distortions. These lead to either slightly different lattice parameter ratios or (mostly) integer multiples,^1^ and/or to somewhat deviating angles between apparently equivalent lattice directions. Fig. 2 ▸ displays some relationships between higher symmetric Bravais lattices using the ratios *a*/*b* and *c*/*b* and the angle γ [which is also used as the monoclinic angle (unique axis **c**)].

If no symmetry and only the lattice metric is known, lattice parameters are defined by *a* ≤ *b*, *i.e.* *a*/*b* ≤ 1. In contrast, *c*/*b* > 0 can have any size, since for the considered Bravais lattices 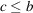 or 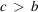 . Like *a*/*b*, γ is also restricted: 0 < γ ≤ 120°, so that the maximum is given by γ/90° = 4/3. For most considered Bravais lattices α = β = 90°, except for *hR* where α = β = γ.

Fig. 2 ▸ is intended to show that small deviations from the highlighted plane (*oP*), displayed arrows (for *tP*, *hP* and *hR*) or single points (*cP*, ‘*cF*’, ‘*cI*’) bear the danger of pseudo­symmetric lattice interpretations. If for the monoclinic angle γ/90° ≃ 1, the lattices are assumed to be *oP*, which changes to *tP* if additionally *a*/*b* ≃ 1. If also *c*/*b* ≃ 1 the lattice is accepted to be *cP*.

Not quite so obvious are pseudosymmetries of centred lattices, as shown by the example of *oC_hP_*, which is an alternative but non-primitive description of *hP*. Since the lattice parameter ratio of *oC_hP_* is described by (*a*/*b*)_o_ = 1/ 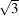 , then also (*c*/*b*)_o_ = (*c*/*b*)_h_/ 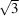 . Equivalent basis vector ratios are illustrated by three example pairs of points along *hP* and *oC_hp_* whose connection leads to 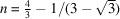 .

For γ ≃ 60° or γ ≃ 109.5° and *a* ≃ *b* ≃ *c*, lattices will be interpreted as primitive descriptions of centred cubic cells.

In fact, the symmetry or pseudosymmetry of a phase is not determined by the lattice metric at all but by the crystal structure. The lattice only has to follow this symmetry but can be higher symmetric. Generally, trigonal phases such as quartz (α-SiO_2_) are represented by the higher-symmetric hexagonal lattice, but, as just discussed, monoclinic phases with γ ≃ 90°, tetragonal phases with *a* ≃ *c* or orthorhombic phases with *a* ≃ *b* ≃ *c* are also considered higher symmetric.

**Figure 3:**
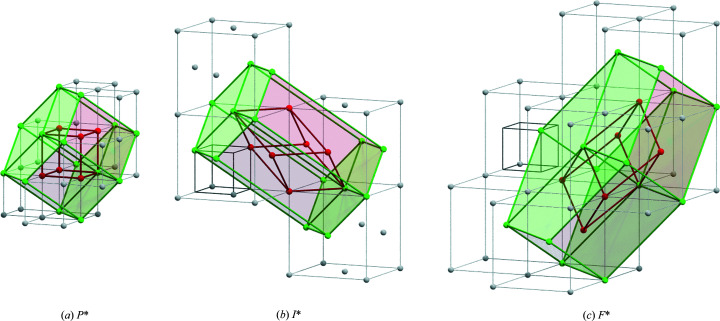
The reciprocal lattices of *cP*, *cI* and *cF*. (*a*) cP* ↔ 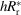 ↔ 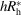 . (*b*) cI* 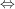 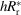 ↔ 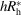 . (*c*) cF* 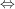 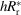 ↔ 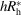 . The equally drawn reciprocal unit cells for *P* 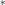 , *I* 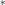 and *F* 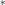 are red in (*a*) but dark grey in (*b*) and (*c*). *I* 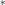 and *F* 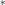 have an incomplete occupation of the corners with reciprocal-lattice points. Therefore, the reciprocal-lattice point arrangement appears like a face-centred lattice for *I* 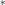 in panel (*b*) but like a body-centred lattice for *cF* 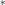 in panel (*c*). The red frame represents in each panel the primitive cell, which is rhombohedral in (*b*) and (*c*). The hexagonal cell (green) results in a primitive superlattice when the red centred reciprocal-lattice points have not been discovered by Kikuchi bands.

*Vice versa*, a phase with *a* = *b* = *c* and α = β = γ = 90° matches to any crystal symmetry except hexagonal (but see Fig. 3 ▸ when lattice points are systematically vacant). However, the probability is very high that not only does the lattice system look isometric but also the crystal system of the corresponding phase is cubic. Unfortunately, the crystal structure as an exclusively relevant physical quantity cannot yet be derived from BKD patterns, despite initial attempts (Lühr *et al.*, 2016 ▸). The structure influence can be very complex, which manifests itself for example in some compounds with numerous polytypes (superstructures) or modifications (Michael & Eades, 2000 ▸; Kogure & Bunno, 2004 ▸; Kogure *et al.*, 2005 ▸; Kościewicz *et al.*, 2010 ▸). Their differences are often minimal in the BKD pattern and of the order of magnitude of the experimental band detection error. At present, such small BKD signal differences can often only be distinguished using comparatively sophisticated pattern matching techniques (Martin *et al.*, 2022 ▸). However, this assumes that the available pattern simulation is unquestionably suitable for this purpose and, furthermore, that other experimental uncertainties can be excluded. Still, this is purely hypothetical, especially in the case of unknown phases and typically unpredictable experimental conditions, like landing electron energy or image distortions in BKD patterns, requiring a correspondingly critical review of the results.

#### 1.1.2. Reciprocal lattice of centred unit cells

The derivation of the lattice metric from a single BKD pattern is based on a correlative analysis of the reciprocal lattice (Li & Han, 2015 ▸; Nolze *et al.*, 2023*a* ▸). For primitive lattices this is comparatively straightforward. Unfortunately, many technically relevant phases are described by non-primitive Bravais lattices, such as ferrite and austenite, *i.e.* α-Fe and γ-Fe, respectively. Since the impact of unit-cell centring on the reciprocal lattice usually receives little attention, it is briefly summarized in Appendix *A* [App appa].

#### 1.1.3. Pseudo-integral absences

For the description of reciprocal-lattice points, Laue indices *hkl* are used which permit any combination of integers for *h*, *k* and *l*. Systematic absences or higher-order interferences reflecting major Kikuchi band edges may indicate another Bravais lattice type if they have the character of integral reflection conditions. Thus, a systematic absence of points in a primitive lattice P* where each unit-cell corner is actually occupied can suggest a centred lattice. However, an F* lattice is also a subset of A*, B* or C*.

This, of course, carries the risk of a pseudosymmetric interpretation of the Bravais lattice type and can have a very significant impact on the lattice parameters and ratios (basis vector definition of superlattices) if Kikuchi bands are systematically left undetected or low-intensity interference orders are ignored, as presently in *CALM* (Nolze *et al.*, 2021 ▸).

Fig. 3 ▸ illustrates an example where any cubic lattice represents a special case of a rhombohedral lattice (red cell). A subset of lattice points even defines a hexagonal sublattice (green cells) if in each cell the red reciprocal-lattice points *hkl* are not described by an equivalent Kikuchi band width. The resulting lattice parameters in Fig. 3 ▸ have, of course, a very specific relationship to the cubic lattice parameter *a*_c_ (Table 1 ▸).

Because of the limited size of the diffraction signal captured by a BKD pattern, but also because of restrictions regarding usable band widths, only a limited part of the reciprocal lattice can be derived. Therefore, there is always a risk of systematically missing reciprocal-lattice points. This risk, along with statistical reasons, is another motivation for finding as many Kikuchi bands as possible.

Summarizing, different aspects can each play a role in the misinterpretation of a lattice type. From a purely theoretical point of view, for a centred cell the symmetry density increases with the number of lattice points per unit cell, *i.e.* even a false Bravais lattice type with identical lattice parameters also represents pseudosymmetry, *e.g.* *F* instead of *P*. Thereby, the reliability of the derived Bravais lattice type is never 100% certain, simply because of calibration limitations or the accuracy of the Kikuchi band positioning (Li & Han, 2015 ▸; Nolze *et al.*, 2021 ▸). In addition, there is the not entirely independent issue of symmetry, which is inherent in each lattice point itself.

#### 1.1.4. Crystal structure

As mentioned already, during phase transformations order/disorder phenomena in the structures also trigger lattice distortions. Thus, practically any change in the crystal structure results in lattice variations. Nevertheless, since the crystal lattice is much easier to monitor than the crystal structure, the lattice parameters serve as *de facto* fingerprints in phase identification.

However, small changes in the crystal structure and lattice are often also associated with changes affecting at least part of the symmetry. Crystallographic subgroup relationships between the initial and resulting crystal structures exist (Müller, 2013 ▸), with some symmetry preserved while some is lost. Derived subgroup diagrams indicate the closest crystallographic relationships (maximal subgroups) between parent and child phases only (Zwart *et al.*, 2008 ▸; Capillas *et al.*, 2011 ▸; Ivantchev *et al.*, 2000 ▸). Because of the strong relationship, simple transformations exist between basis vectors that allow a description of the unit cell according to the new symmetry or sublattice (Hornfeck & Harbrecht, 2009 ▸). Unfortunately, the group–subgroup concept is not generally applicable. Simple examples are metal structures like Fe. The transformation of the face-centred cubic (f.c.c.) into a body-centred cubic (b.c.c.) phase is explained by a noticeable shearing of the structure. The relationship between adjacent atoms during the transformation does not get lost, but their coordination number reduces from 12 to 8, which is accompanied by a 4% contraction in the atomic distance. This also requires a complete realignment of the symmetry elements, although the (point-group) symmetry before and after the transformation appears to be the same, and the symmetry density also remains practically unchanged if one disregards the volume reduction of −1%.

#### 1.1.5. Crystal symmetry

For discrimination between phases, symmetry in BKD patterns can be used (Dingley & Wright, 2009 ▸; Peng *et al.*, 2021 ▸), but only if their symmetries are clearly different from each other. An unambiguous proof of actual symmetry in experimental BKD patterns is only possible if the lattice does not give rise to any suspicion of pseudosymmetry. With an unknown phase this cannot be ruled out at all and measurement uncertainties are unavoidable.

### 1.2. Imaging and other errors

In addition to phase-specific excess-deficiency effects, experimental BKD patterns can be affected by local distortions due to detector optics or by electromagnetic interference fields. These do not have to occur in the selected imaging field but can be invisible to the observer somewhere on the sample, around the sample in the scanning electron microscope or even caused by the EBSD detector itself. Such patterns cannot be perfectly processed since they do not match the assumed purely gnomonic projection of the signal.

In contrast, physics-based BKD pattern simulations appear perfect. However, they have also been shown to reflect experimental patterns adequately only in terms of quality. More detailed analysis reveals that the electrons that actually contribute to the pattern have a slightly lower energy than the primary electrons assumed in the simulation. In addition, this effective energy varies very slightly with the scattering angle (Winkelmann *et al.*, 2019 ▸).

## 2. Software used

For the physics-based BKD pattern simulations, the program *DynamicS* (Bruker Nano, Berlin, Germany) has been used (Winkelmann *et al.*, 2007 ▸). Diffraction ray tracing in *DynamicS* is based on a square grid on a cube surface. Correctly assembled, the projection cube satisfies the requirements of all relevant symmetries [see *e.g.* Nolze (2013 ▸)].^2^

In the present investigations, about a thousand *hkl* were considered for each phase in the simulation of the master patterns. An electron energy of 20 keV was used for the relativistic calculation of the electron wavelength. For simplicity, a single plane of the projection cube was analysed, representing ∼17% of the total master pattern. It displayed the standard projection, *i.e.* [001] was always located at the pattern centre. The crystallographic description of the lattice plane traces has been derived from the definition of the coordinate systems (lattice parameters used for the simulation, projection centre, crystal orientation). The trace positioning and band profile extraction are described for example by Day (2008 ▸) and Nolze *et al.* (2021 ▸, 2023*a* ▸).

For the determination of the lattice parameters *CALM* (Version 1.5.2) was used, which exclusively evaluates the angular position of the strongest extrema of the first derivative of the band profiles (Nolze *et al.*, 2021 ▸). For the extrema determination smoothing level 7 was chosen. Only bands whose band widths *W*_*hkl*_ are in the range of 4 ≤ *W*_*hkl*_ ≤ 9° are considered for the determination of the mean lattice parameter and the standard deviation. Since the trace positions of the diffracting {*hkl*} were calculated and the PC was known, both can be ruled out as even partial causes of the deviations discussed below.

## 3. Results

In the present analysis, simulated BKD patterns of about 350 phases were studied. The selection of phases is not representative, nor can it be. They were specifically chosen to assess the limitations of determining lattice parameters when analysing individual BKD patterns in *CALM*. Thus, phases with particularly small or comparatively large lattice parameters or lattice parameter ratios were selected, as were phases with heavy, light or very similar element combinations. Phases with an ordered structure or pseudosymmetric lattice were also considered. These difficult conditions describe rather exceptional situations and are intended to point out the limitations that other experimental techniques such as XRD also have, albeit to a different extent. For example, the crystal lattice of troilite (FeS) as derived by *CALM* is the same as that initially described by Alsén (1925 ▸). This outdated crystal structure description is still listed in current databases, although later XRD analyses discovered an element ordering which caused a multiple size of the unit cell: *a* = |**a**_1_ + **a**_2_| = 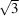 *a*′, *c* = 2*c*′ (Bertaut, 1954 ▸).

### 3.1. Binary compounds with simple structures

The current approach in *CALM* of using only bands that are uniquely identifiable by more or less symmetric band edge positions, ignoring unclear superpositions and not caring about the specific interference order of a band edge, reduces the uncertainty of a solution. However, it also bears the risk of deriving a pseudolattice by neglecting lower interference orders. Reciprocal-lattice points are then systematically omitted or an insufficient number of bands are considered during the analysis. A supercell in reciprocal space results, which is the equivalent of a smaller unit cell in real space.

For element structures, assigning a pseudosymmetry is practically impossible. For compounds, especially where the atomic positions are identical to the lattice point positions, it is quite conceivable. The structure types *B*1 (NaCl) and *B*2 (CsCl) show an increased susceptibility to misinterpretation of the Bravais lattice type. As proof, Table 2 ▸ lists 52 phases with *B*1 and 35 phases with *B*2 structure type.

As Table 2 ▸ shows, *CALM* does not extract the correct Bravais lattice type for almost half of the phases. For *B*1 a *P* lattice with halved lattice parameter is derived. For *B*2, instead of the correct *P* lattice an *I* lattice results, but with identical lattice parameter (see also Table 3 ▸). Both false solutions have twice as many lattice points, *i.e.* in reciprocal space certain lattice points remain systematically undetected.

### 3.2. More complicated structures

Using automatic band width detection in *CALM*, 28 of 144 phases characterized with crystal structures more complicated than *B*1–*B*4 were described by a pseudosymmetric lattice (Table 3 ▸). In practice, both reasons for pseudosymmetry come into play.

Minimal distortion of the lattice while preserving the lattice point density (*m* ≃ 1) requires a very precise description of the trace positions and the position of the PC, *cf.* the pseudocubic or pseudotetragonal Sb_2_S_3_, CoAsS and Fe(OH)_3_. This increases the symmetry of the lattice system but keeps the Bravais lattice type: *oP* → *cP*. More frequently, as for the already discussed *B*1 and *B*2 structures, an apparently higher number of lattice points are found, which increases the lattice point density because of the smaller unit-cell volume. In Table 3 ▸ these are phases with *m* > 1.

It is more common than expected that phases still carry a relationship to higher-temperature modifications whose symmetry is usually higher. Orthorhombic phases such as Mo_2_C or Ni_3_Nb, which show only a minimal deviation from a hexagonal metric, are typical representatives. Such phases are often identified in *CALM* by their higher-symmetric counterpart. For simulated patterns, a known PC and calculated trace positions, acceptable maximum deviations can be reduced to minimum values (0.1%), which can rule out the hexagonal solution. However, for experimental patterns with excessive-deficiency effects, significant noise, an imperfect PC and refined trace positions, maximum deviations should not realistically be less than 1%. Moreover, for polytypes such as Co_3_W, discussed by Nolze *et al.* (2017 ▸), it turns out that *m* need not necessarily be integer as Table 3 ▸ suggests.

In summary, the danger of deriving a pseudosymmetric solution for a lattice from a single wide-angle BKD pattern is definitely present, even if the correct lattices were determined for the majority of the phases studied. We assume that the risk of finding superlattices is much higher than when using selected-area electron diffraction (SAED) in transmission electron microscopy (TEM), mainly because of the superimposing bands in BKD patterns. Superstructure reflections, which appear as weak spots between main reflections in SAED, are at most guessable in a BKD pattern. For instrumental reasons, of course, the risk of finding superlattices is also higher than for XRD, whose reflection splitting should be better by about two orders of magnitude despite superposition. Nevertheless, BKD patterns provide very fast and convenient access to the translation lattice, where possible pseudosymmetries can be excluded with additional analyses in TEM. However, it should be remembered that the derived lattices can be converted into each other relatively easily, since either points are systematically missing or individual lattice points have been determined erroneously. The majority of the reciprocal-lattice points, on the other hand, are correctly derived. They are a subset of the true lattice.

## 4. Discussion

### 4.1. Pseudosymmetry in BKD patterns

Since in typical BKD patterns more than 85% of the total signal is not captured and, in addition, information is superimposed, it cannot be completely ruled out that this significantly affects the description of the extractable crystal lattice. Superposition is difficult to predict or interpret since several factors influence the quality and quantity of extractable data. The crystal structure containing all atomic coordinates [*x*_*j*_, *y*_*j*_, *z*_*j*_] within the unit cell defines the structure factor, 

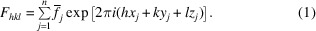

It controls the kinematic intensity *I*_*hkl*_ of an interference *hkl* which is, for BKD patterns, proportional to the structure amplitude (Winkelmann *et al.*, 2016 ▸),

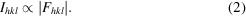

For phases described by an invariant set of atomic positions [*x*_*j*_, *y*_*j*_, *z*_*j*_], as in structure types like NaCl, CsCl, ZnS (zincblende) or CaF_2_, the exponential term is identical. From this one might conclude that the BKD patterns of all isostructural phases should look very similar, but this is only true to a rough approximation. There are several parameters that determine the visual appearance of Kikuchi bands. First, the translation lattice in combination with the electron energy controls the band widths and thus the purely geometric superposition of bands. In combination with the limited size of the captured sector, this leads to an asymmetric position of the band edges. For the first derivatives of a considerable number of bands θ_asym_ = |θ_min_| − |θ_max_| ≠ 0 holds. Kikuchi bands are not considered in the lattice parameter analysis if |θ_asym_| > 0.2° or the extracted band width deviates by more than 0.2° from the geometrically predicted value (Nolze *et al.*, 2023*a* ▸). Second, the chemical composition in terms of the atomic scattering factor *f* affects the band edge profile. The smaller the mean 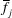 , the sharper the band edges appear [Part II of this series, Nolze *et al.* (2023*b* ▸)]. Third, larger 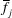 increase the overall intensity. However, this does not necessarily lead to a better processable diffraction signal, since the background formed by numerous higher-indexed bands with appreciable intensity also increases (Nolze *et al.*, 2023*b* ▸).

Regardless of the specific reason, when band edge profiles effectively become more blurred, the precision and accuracy of the Bragg angles, and thus of the lattice parameters, deteriorate. This reduces the chance of detecting small distortions or more narrow but weaker bands caused by superstructures.

### 4.2. Atomic scattering factors

Systematically absent reciprocal-lattice points can be explained plausibly as a result of either overly similar scatterers – *e.g.* Sr and Zr in SrZrO_3_ or Ba and Sn in BaSnO_3_ – or overly dominant scatterers – *e.g.* in SnO_2_ (cassiterite) or PtB. Both suggest an apparently higher lattice point density.

#### 4.2.1. X-ray scattering

Given the high importance and widespread use of X-ray diffraction, knowledge of the scattering of X-rays by atoms and ions is one of the fundamentals in materials science education. Therefore, this aspect will be briefly recalled for comparison.

In Vol. C of *International Tables for Crystallography* the X-ray scattering power of elements is listed either in tabular form or by a parameter description using *a*_*i*_, *b*_*i*_ and *c*_*i*_, valid for 0 < *s* < 2 Å^−1^ (Prince, 2004 ▸; p. 578): 

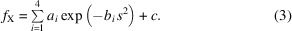

**Figure 4:**
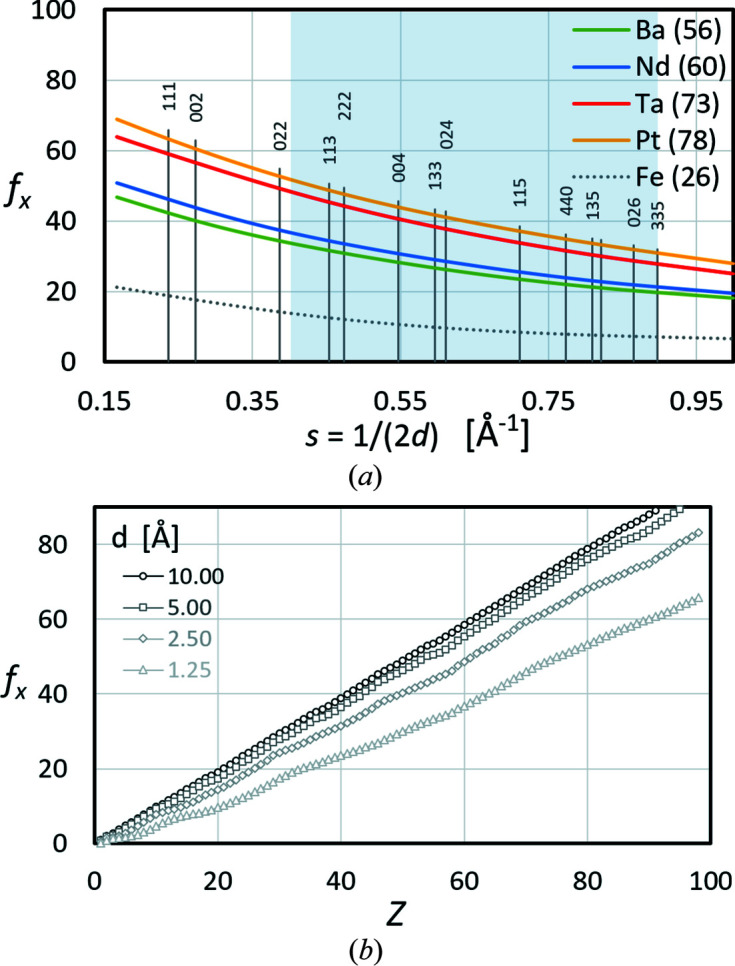
X-ray atomic scattering factors *f*_X_ as derived by Cromer & Mann (1968 ▸). (*a*) The change in *f*_X_ with *s* or the interplanar distance *d*. The positions of the indexed vertical bars indicate *s* = sinθ/λ = 1/(2*d_hkl_*) for γ-Fe. (*b*) A plot showing that *f*_X_ ∝ *Z* for constant *s*. The selected *d* represent *s* < 1 (0.05, 0.1, 0.2 and 0.4 Å^−1^), which represent common band widths in BKD patterns.

Equation (3) describes a monotonic decrease in *f*_X_ with *s*, as shown for example for a few elements in Fig. 4 ▸(*a*). The graphs are only displayed for the range of 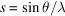 which is relevant for the interpretation of Kikuchi bands (2 < ½*W_hkl_* < 4.5°), highlighted in light blue. The vertical lines indicate discrete *s* = 1/(2*d_hkl_*) < 0.9 of all possible *hkl* for γ-Fe. Note that *s* is directly proportional to the angular width of the Kikuchi bands. The light-blue highlighting indicates that 111, 002 and 022, and also bands which are wider than 335, are excluded during lattice parameter determination in *CALM*.

If one considers all elements as in Fig. 4 ▸(*b*), between *f*_X_ and *Z* an approximate proportionality exists. For *s* = 0 [not displayed in Fig. 4 ▸(*a*)] the atomic scattering factor for X-rays is equal to the number of electrons. For elements this means *f*_X_(0) = *Z*. The higher *s* – or according to Bragg’s law the smaller the given *d* = 1/(2*s*) – the lower *f*_X_ becomes. The direct proportionality between *f*_X_ and *Z* remains roughly unchanged.

In summary, it follows that the different scattering factors allow elements to be distinguished from one another. Large differences in the atomic number result in significant differences in the scattering power, while small differences indicate elements with similar atomic numbers.

#### 4.2.2. Electron scattering

Vol. C of *International Tables for Crystallography* also lists in Table 4.3.2.2 on p. 282 parameters for calculating elastic atomic scattering factors for electrons [Prince (2004 ▸); note that there are other parameterizations besides the one used, *e.g.* Kirkland (2020 ▸) and Lobato & Van Dyck (2014 ▸)]. They refer to the original approach presented by Peng *et al.* (1996 ▸) [with the wrong parameters for Tb corrected by Prince (2004 ▸)] which is based on a similar parameterization as given in (3), but with *i* = 1–5 and no *c*: 

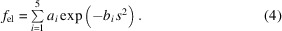

**Figure 5:**
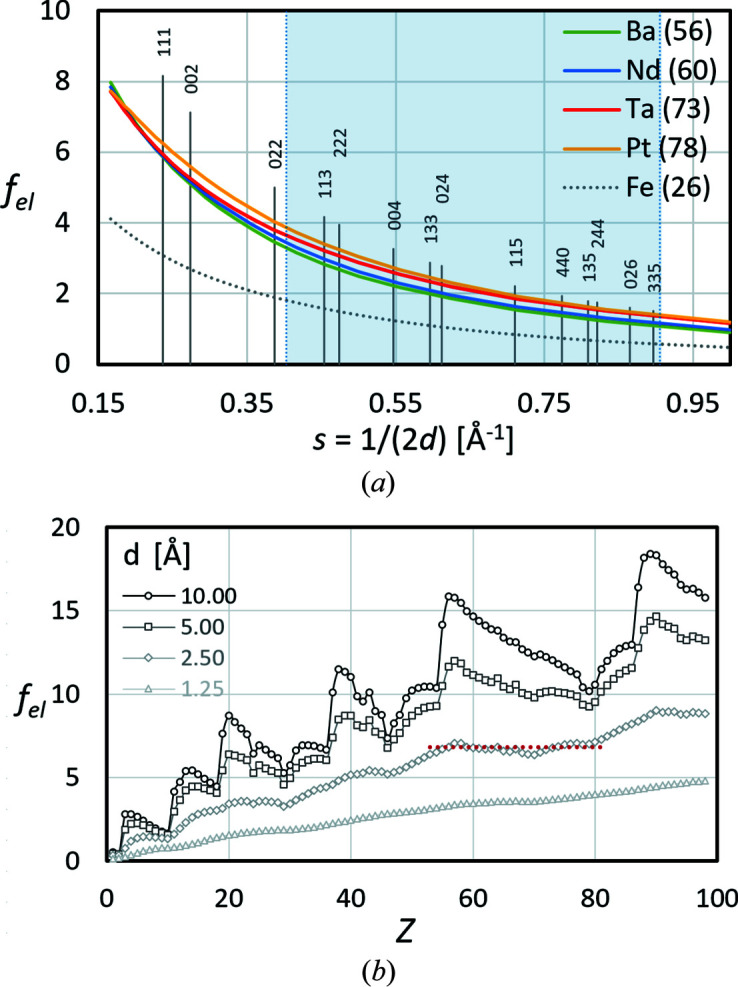
Electron atomic scattering factors *f*_el_ as derived by Peng *et al.* (1996 ▸). (*a*) The change in *f*_el_ with *s* = sinθ/λ = 1/(2*d_hkl_*). Since for small Bragg angles 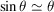 holds, the numerical value for *s* for the indexed vertical bars (γ-Fe) also gives about one-tenth of the Kikuchi band width for 20 keV electrons in degrees. (*b*) A plot of *f*_el_ = *f*(*Z*), proving that *f*_el_ ∝ *Z* can be assumed only for *d_hkl_* > 1.25 Å (*s* > 0.4 Å^−1^). The red dotted line indicates a large *Z* range for *d* = 2.5 Å where *f*_el_ is nearly constant.

For the same elements as in Fig. 4 ▸(*a*), the expected decreasing curve plots of the scattering factors with increasing *s* are shown in Fig. 5 ▸(*a*). However, in contrast to *f*_X_ the electron scattering factors *f*_el_ of Ba to Pt (*Z* = 56–78) differ only insignificantly, *cf.* also the red dotted line for *d* = 2.5 Å in Fig. 5 ▸(*b*).

The peculiar *f*_el_ jumps^3^ for larger *d*_*hkl*_ are explained by the *Z* dependence of the atomic radius (Yamashita *et al.*, 2018 ▸). Fortunately, as *d*_*hkl*_ decreases, the jumps smooth out more and more until *f*_el_ becomes almost an only monotonically increasing curve as for *f*_X_. Only for narrow but often high-intensity bands of low-indexed (*hkl*) might this effect become relevant.

Nevertheless, it seems much more difficult to distinguish elements with similar atomic number in a phase by electron diffraction, especially in BKD patterns and with the simple band edge determination approach used in *CALM*. This will lead to pseudosymmetric interpretations of the crystal lattice, despite the exclusive use of wider band widths only. According to 

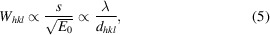

a lower electron energy *E*_0_ would increase the band widths even for higher *d*_*hkl*_, but the band edge detection becomes less reliable and, more importantly, the number of detectable bands drastically reduces the statistical relevance of the derived lattice parameters (Nolze *et al.*, 2023*a* ▸). In any case, there remains at least the concern that the monotonicity of *f*_el_ is lost for the band width range considered with falling electron energy, which tends to favour a pseudosymmetric interpretation of the derived reciprocal-lattice point arrangement.

### 4.3. Structure amplitude

#### 4.3.1. Element structures

From equations (1) and (2) it follows that for element structures the intensity of *hkl*, if different from zero, scales with the atomic scattering factor of the element (Table 4 ▸). For the simplest structure types, f.c.c. and b.c.c., the atomic scattering factor is only multiplied by the number of lattice points per unit cell. Figs. 4 ▸ and 5 ▸ also indicate that for these simple structure types it follows that the higher the interference order the lower the intensity.

For more complicated element structures (structure types *Ax* with *x* ≥ 3), where more than a single atom is described by a lattice point, various rules for |*F*_*hkl*_| result for different combinations of *h*, *k*, *l* which again contain as a factor the number of lattice points per unit cell. In Table 4 ▸, hexagonal closed packed (h.c.p.) or diamond are most prominent. A comprehensive compilation of crystallographic prototypes containing other element structure types can be found in the work of Mehl *et al.* (2017 ▸) and Hicks *et al.* (2019 ▸, 2021 ▸).

The occupation of exclusively special positions in the unit cell leads to few uniform |*F*_*hkl*_| calculation formulas in Table 4 ▸. The presence of two or three explicit formulas means that there are only two or three vertically shifted proportional |*F*_*hkl*_| curves for an element, which decay with increasing *s* similar to one of the *f*_el_ curves displayed in Fig. 5 ▸(*a*). Therefore, for element structures, pseudosymmetry misinterpretations might arise only from minimal distortions of the lattice, as illustrated in Fig. 2 ▸.

#### 4.3.2. Binary compounds of type *AB*

Considering binary compounds with 1:1 stochiometry, differences occur in addition to sums of atomic scattering factors (Table 5 ▸). Since a lattice plane has several interference orders, for example in the case of structure type *B*2, then the intensity of an odd order *hkl* with *h* + *k* + *l* = 2*n* + 1 is determined by the difference between the atomic scattering factors, while all even orders are described by their sum. Therefore, for element combinations with very similar *f*_el_ odd interference orders are difficult to detect in BKD patterns of phases with the *B*2 structure. Similar conditions also exist for phases with the *B*1 structure type.

In contrast, no pseudosymmetry was detected for 16 binary compounds with the zincblende structure (*B*3, *hP*) or for eight with the wurtzite structure (*B*4, *cF*). The reason is that the corresponding element structure types of Mg (*A*3, *hP*) and diamond (*A*4, *cF*) have the same lattice types.

### 4.4. The impact of global extrema for band width detection

**Figure 6:**
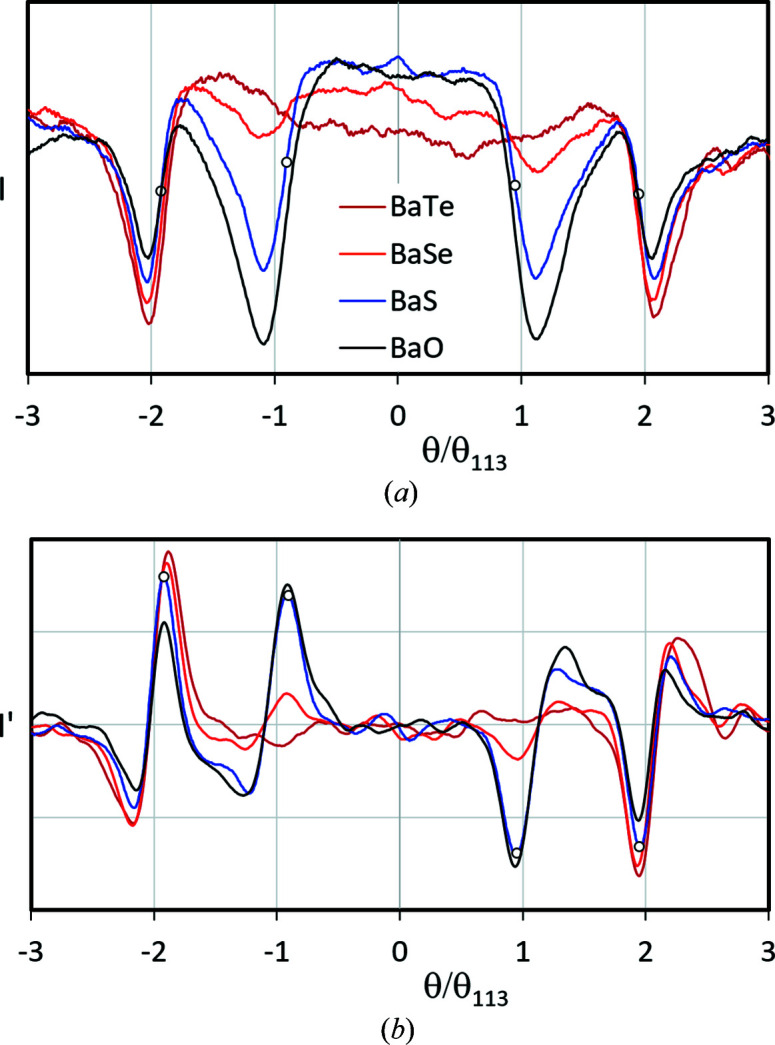
(*a*) Band profiles and (*b*) their first derivatives of one and the same (113) from simulated BKD patterns for five different Ba compounds with the *B*1 structure. Using θ/θ_113_ as the abscissa normalizes the band widths for all phases. The integers indicate the interference orders. The profiles in panel (*a*) vary mainly with the atomic number of the second element.

One of the drawbacks of the first derivative used for band edge detection in *CALM* is that only the global extrema are currently applied. In Fig. 6 ▸, the resulting consequences are shown by comparing (113) band profiles derived from simulated BKD patterns of five Ba compounds with the *B*1 structure.

The accumulated band profile intensity *I* in Fig. 6 ▸(*a*) shows that with decreasing Δ*Z* = |*Z*_Ba_ − *Z*_*B*_| the first interference orders at θ/θ_113_ = ±1 diminish. Therefore, *CALM* recognizes along the reciprocal-lattice direction [113]* as the first reciprocal-lattice point 226, since 113 is either of minor intensity or practically invisible.

**Figure 7:**
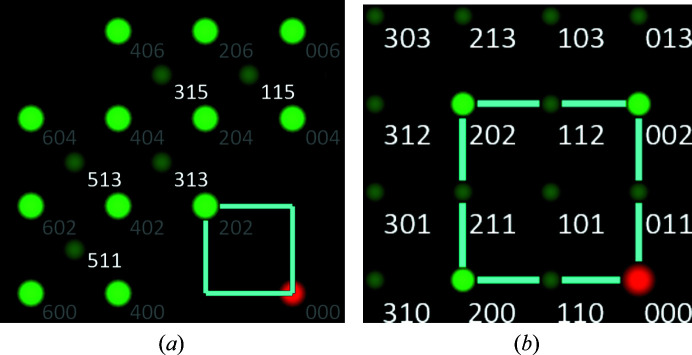
Derived reciprocal-lattice points *hkl* from (*a*) a *B*1 phase and (*b*) a *B*2 phase indicating a false lattice description, projected along [010]*. The indices only distinguish between reciprocal-lattice points with *k* = 0 or *k* = 1 in order to illustrate the layered arrangement. The distribution in panel (*a*) describes an I* lattice. Because of the systematic lack of *hkl* in panel (*b*), the actual P* lattice is misinterpreted as an F* (*I*) lattice, *cf*. the incorrect unit-cell edges drawn by the turquoise frame. Note that centred reciprocal-lattice points are shown smaller and darker than those sitting at the corners of the assumed unit cell.

The reciprocal-lattice point distribution derived from all discovered band profiles and projected along [010]* is shown for BaO as an example in Fig. 7 ▸(*a*). If not all first interference orders *h*1*l* described by the dark-green (centred) reciprocal-lattice points are discovered, the actual reciprocal F* lattice is only described by the light-green points, which suggest a P* lattice having half the lattice parameter compared with *a* of the true *F* lattice. Since narrow bands are excluded because of their limited accuracy, reciprocal-lattice points close to the red origin are missing in Fig. 7 ▸(*a*). However, from translation symmetry it follows that there must also be centring lattice points at these empty locations.

For the Ba compounds compared in Fig. 6 ▸(*b*) an unquestionable characterization only follows for BaO. For BaS the global minimum (right) indicates the edge of band 113, but the global maximum (left) marks the band edge of 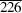 , which is slightly stronger than 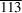 . During automated processing, *CALM* ignores bands with such asymmetric descriptions. Fortunately, there are other bands in BaS which only have a visible first order and deliver centred lattice points. The band profile of BaSe in Fig. 6 ▸(*b*) shows slight intensity minima at 113, but its first derivative in Fig. 6 ▸(*b*) provides only weak extrema. They are ignored in the automatic band edge search currently used. Nevertheless, the band edges for BaS and BaSe can be corrected manually by assigning the automatically found ones to a higher interference order. However, for BaTe, regardless of the Kikuchi band chosen, there is no evidence of a lower interference order and thus of a superlattice. The consequence is a unit cell whose edge length is only half the size of the real one.

Fig. 7 ▸(*b*) illustrates the reason for misinterpretation of the lattice for phases with the CsCl structure. The systematic absence of reciprocal-lattice points turns a P* lattice into an I* lattice. In principle, in each layer of the reciprocal lattice (at least) every second point is missing.

### 4.5. Lattice or sublattice?

In addition to small lattice distortions as for the phases in Table 3 ▸ with *m* = 1, the evaluation of BKD patterns adds the problem that reciprocal-lattice points are systematically not recognized. This happens when the corresponding Kikuchi bands are too weak or cannot be reliably detected for other reasons. The result is always a perfectly fitting reciprocal sublattice, suggesting a higher lattice point density in the crystal lattice. Typical examples are ordered or disordered structures, heavy elements with apparently or actually higher site symmetry, or simply phases whose constituents scatter similarly.

**Figure 8:**
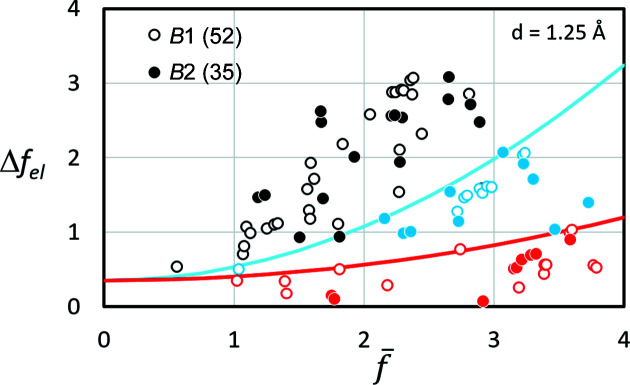
The difference in the scattering power Δ*f*_el_ between the two elements in a binary compound for 52 *B*1 phases and 35 *B*2 phases as a function of the mean scattering power 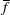 . *f*_*A*_ and *f*_*B*_ are computed for *d* = 1.25 Å which is equivalent to *s* = 0.4 Å^−1^. This represents the narrowest bands considered in *CALM* for 20 keV electrons. Black symbols indicate phases where the extrema of the first derivative yield the correct lattice type. Blue symbols represent phases where further interference orders were evident, leading to the correct lattice type. Red symbols illustrate phases where no indication of the correct superlattice existed.

In order to assess better the influence of different scatterers, 52 phases with the NaCl structure (*B*1) and 35 phases with the CsCl structure (*B*2) have been investigated as examples. For these binary phases of simplest stochiometry *AB*, the difference in scattering power Δ*f*_el_ = |*f*_*A*_ − *f*_*B*_| can be drawn as a function of the mean scattering power 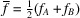 (Fig. 8 ▸).

As expected, phases with comparatively small Δ*f*_el_ generate a Kikuchi signal which hides the true lattice and suggests a pseudosymmetric lattice (superlattice) with a higher lattice point density. This means that the correct solution is a subgroup of the discovered lattice. These phases are represented by red symbols.

Only the black symbols in Fig. 8 ▸ indicate phases for which the correct lattice description results immediately, even with a fully automatic search of band positions and widths. When automated band width detection is used, the blue element combinations drawn in Fig. 8 ▸ also result in a pseudosymmetric superlattice. However, a manual correction of those bands which display lower interference orders of weak intensity enables a correct description of the Bravais lattice type and the corresponding unit-cell dimension.

Since the three regions in Fig. 8 ▸ do not appear to overlap, a purely empirical separator line can be defined. It follows an analytical description using *x* = 4 for the red and *x* = 2 for the blue line: 

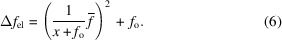

Unfortunately, both *x* and the offset *f*_o_ at 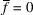 are dependent on the selected *d* or *s*.

**Figure 9:**
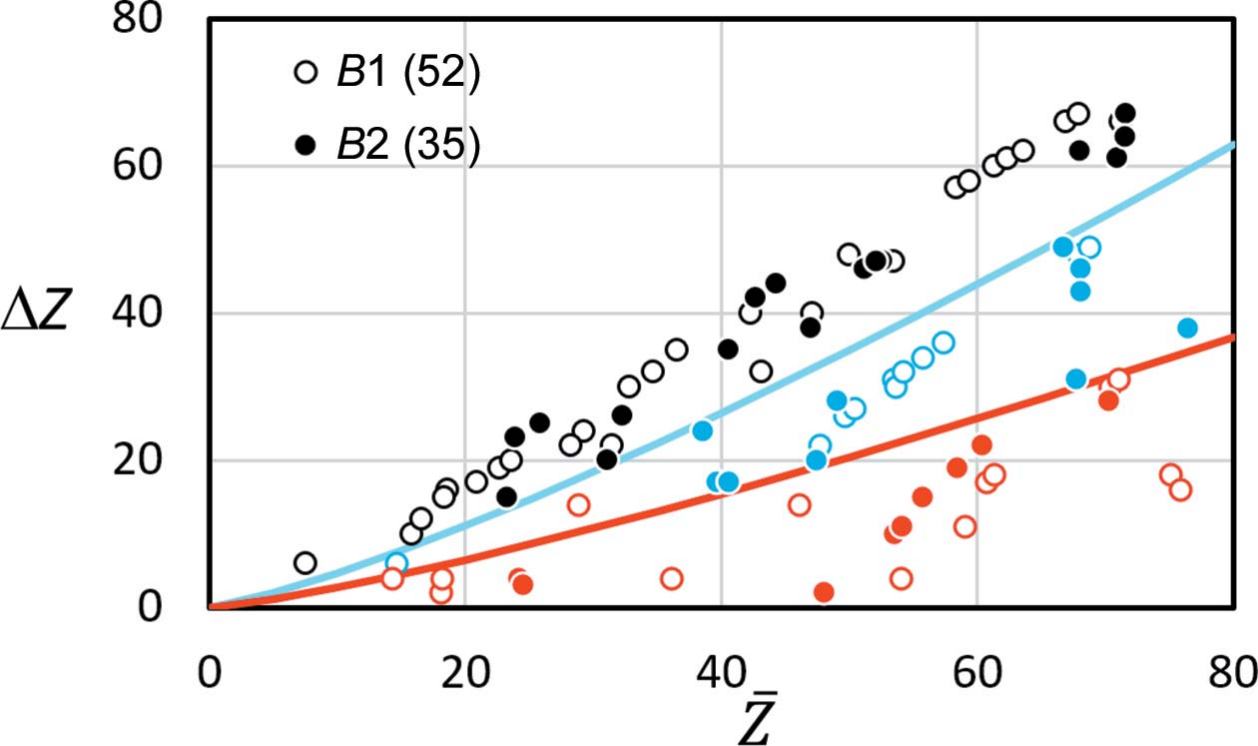
The difference in the atomic number Δ*Z* between the two elements in a binary compound as a function of the mean atomic number 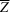 . As in Fig. 8 ▸, black symbols indicate phases where the extrema of the first derivative yield the correct lattice type. Blue symbols represent phases where further interference orders were evident, leading to the correct lattice type. Red symbols illustrate phases where no indication of the correct superlattice existed. The red and blue lines are proposed to separate correct from pseudosymmetric solutions.

Therefore, a correlation with the atomic number *Z*, which is defined independently of *d* and *s*, seems more appropriate. In Fig. 9 ▸, the difference in the atomic numbers weighted by the mass fraction *c*_*i*_, 

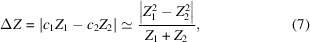

is plotted against the mean atomic number estimated by 

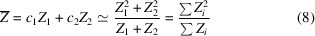

(Müller, 1954 ▸; Lloyd, 1987 ▸). The colours used for the symbols in Fig. 9 ▸ have the same meaning as in Fig. 8 ▸.

For the purely empirical red line (*y* = 6.5) and blue line (*y* = 3.8) drawn as separators between the black, blue and red symbols we propose 

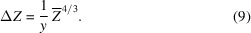

As the single red *B*1 symbol for CaSe in the blue region at 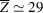 shows, equation (9) does not always provide a reliable prediction. However, Kikuchi band profiles of 430 (indexed for a pseudosymmetric unit cell with half the lattice parameter) in BKD patterns of CaSe indicate an order with half the Bragg angle, so the red symbol could also be blue. This shows how sensitively the lattice description sometimes depends on individual Kikuchi bands.

In summary, the examples in Figs. 8 ▸ and 9 ▸, and especially those in Table 3 ▸, show that pseudosymmetry can become a significant problem in the crystal lattice interpretation of individual BKD patterns. Even if the crystal system has been correctly discovered, there is a probability that the Bravais lattice type is wrong. There is little recourse to rules based on the elements contained in the phases. The only certainty is that elements with very similar atomic numbers are, as expected, hardly distinguishable and favour a pseudosymmetric description of the lattice. However, this does not only concern directly neighbouring elements in the periodic table, as the symbols marked in red prove. As a first approximation, for the *AB* phases considered here, the distance between the elements should be at least 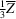 . A distance of 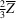 seems to be safe.

## 5. Summary and conclusions

In the present work, the occurrence of pseudosymmetric solutions during interpretation of the crystal lattice from individual BKD patterns and possible reasons for it have been discussed. Due to the erroneous determinability of existing diffraction features, both lattice and reciprocal-lattice uncertainties arise, which can only be minimized. In order to exclude experimental influences as far as possible, simulated BKD patterns of about 350 phases have been used to estimate the feasibility limits. The lattice parameter ratios and angles can be reproduced exactly as expected when using calculated band positions in combination with a perfect projection centre. Only for phases whose lattice deviation is smaller than a declared maximal deviation is a higher-symmetric interpretation preferred as the solution.

The analysis shows that deviations occur even under perfect conditions for simulated BKD patterns, a known projection centre and calculated trace positions of lattice planes. The limited size of a simulated BKD pattern always leads, purely mathematically, to a symmetry reduction in the position, width and shape of the band profiles. If one imagines further experimental influences such as excess deficiency, a highly accurate but not exact projection centre, erroneous band positions due to image noise *etc.*, the deviations increase further and require a critical error discussion. This raises the question of whether the deviations are the result of a true symmetry degradation or are simply caused by experimental or analytical limitations. Regardless, even with the primitive approach presented here, the resulting errors are significantly lower than predicted in the past (Dingley & Wright, 2009 ▸).

On the other hand, crystal symmetry even in simulated BKD patterns gets lost, *i.e.* symmetry-equivalent bands are not congruent and can look very different, to the point of being unrecognizable. This means that a lack of perfect symmetry is of only limited significance.

Analysing isostructural phases, it is noticeable that their susceptibility to inherent pseudosymmetry shows a correlation with chemical composition. The reason is the atomic scattering factor, which is much less sensitive for electrons than for X-rays and as a function of *Z* does not grow monotonically with 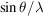 . Fortunately, Kikuchi bands of {*hkl*} with large lattice plane distances are often not suitable during lattice parameter determination because of misleading band widths.

To investigate the discriminability of different elements, the BKD patterns of phases with NaCl (*B*1) and CsCl (*B*2) structures were analysed. It was found that for a significant fraction only a pseudosymmetric lattice can be derived. The resulting higher lattice point density delivers either a halved lattice parameter (*B*1) or an additional centring (*B*2). Although it can be shown that there is a correlation with chemical composition for the binary *AB* compounds studied, this cannot be transferred to other substance classes.

Despite all the drawbacks, it should be clear that a crystal lattice derived from a single wide-angle BKD pattern, even if it is not completely correct, still represents part of the true solution. The crystal lattice found must be at least a sublattice of the true lattice. Therefore, either the lattice parameters are correct, although the true lattice has further centrings, or the true basis vectors are a linear combination of the found basis.

## Figures and Tables

**Figure 10:**
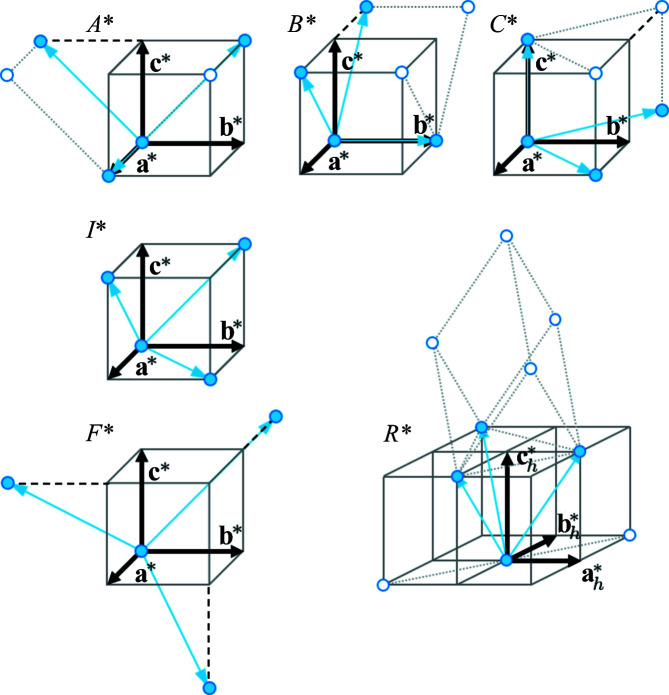
Unit cells of centred lattices in reciprocal space. Assuming the unit cell to be a parallelepiped defined by the basis vectors a*, b* and c*, for A*, B*, C* and I* only half of all the unit-cell corners, for R* only one-third and for F* only a quarter are occupied by lattice points. The shortest translation vectors t* [blue arrows between (filled) lattice points] are commonly not parallel to the basis vectors.

**Table 1:** Rhombohedral and hexagonal descriptions of the reciprocal lattices of cubic Bravais lattice types The derived lattice parameters refer to the cubic *a*_c_ parameter.

Lattice	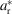	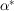	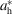	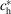
cP*	1/*a*_c_	90°	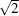 /*a*_c_	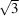 /*a*_c_
cI*	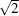 /*a*_c_	60°	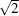 /*a*_c_	2 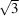 /*a*_c_
cF*	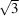 /*a*_c_	109.5°	2 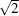 /*a*_c_	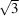 /*a*_c_

**Table 2:** Bravais lattices derived in *CALM* for phases with *B*1 and *B*2 structures Single lower interference orders *n* are either invisible, visible but not automatically detectable, or automatically detectable leading to the correct lattice.

	Wrong Bravais lattice		
	Lower *n* invisible	Lower *n* visible	Correct Bravais lattice
*B*1	KCl, BaTe, GdSb, ErSb, TmSb, PbTe, BiTb, BiHo, BoTe, CaS, CsSe, SrTe, SrSe, MgS	NaCl, BaSe, PrAs, NdAs, GdAs, GdSc, TbAs, HoAs, TmAs, PbSe, BiSe	FiF, KF, CaO, TiC, TiN, MnO, CoO, NiO, NaBr, ZrO, NbC, CdO, CaTe, BaO, BaS, EuS, GdN, TbN, HoN, ErN, YbO, HfC, TaC, PbS, SrS, SrO, MgSe

*B*2	FeTi, FeV, AgIn, CdCe, CdPr, AgSm, DyAg, AgTm, TiI	CuPd, PdTi, AgZn, CsBr, ZnCe, AuZn, AuCd, TlBr, SrTl, ThTe	BeCo, NiAl, BeCu, RbCl, MgSr, BePd, AgMg, LiAg, CsCl, MgCe, MgPr, AlNd, OsSi, AuMg, TlCl, CaTl

**Table 3:** Pseudosymmetric Bravais lattices found during the analysis of simulated BKD patterns The found Bravais lattice is given with respect to the true lattice parameter and Bravais lattice type. ½*a* means that the found lattice parameter *a* is half the true one. However, for orthorhombic phases *a* can also be *b* or *c* and is therefore given in parentheses after the phase name. The last column indicates that the lattice point density is *m* times higher due to pseudosymmetry.

Found	True		Phases	*m*
*oP*	½*cP* *a*	*oP*	Al_2_SiO_4_ (*c*), V_2_C (*b*), FeSi_2_Ti (*c*)	2
*oS* †	½*cP* *a*	*oP*	Ni_3_Nb (*b*), Mo_2_C (*b*)	4
*oS*		*oP*	KPrF_4_ (*b*), PbCO_3_ (*a*)	2
*tP*	¼*cP* *a*	*tP*	Al_5_Ti_3_	16
*tP*		*oP* ‡	Sb_2_S_3_	1
*tI*		*tP*	SnO_2_	2
*hP*	(1/ 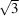 )*a* §	*hP*	W_2_C, FeS	3
*hP*	½*c*	*hP*	PtB	2
*cP*		*oP* ¶	CoAsS	1
*cP*	½*a*	*cP*	Ca_2_TiSiO_6_	8
*cP*	½*a*	*cI*	Ca_3_Te_2_Zn_3_O_12_	4
*cP*	½*a*	*cF*	Some *B*1 structures	2
*cI*		*oI* ††	Fe(OH)_3_	1
*cI*		*cP*	Some *B*2 structures, SrZrO_3_, BaSnO_3_, SnTaO_3_	2
*cI*	½*a*	*cI*	YCu_3_Mn_4_O_12_	8
*cI*	½*a*	*cF*	AlCu_2_Mn, GaMn_2_V	4
*cF*		*cP*	Na_3_OCl, Cu_2_O, Mn_3_InC, FePt_3_, PbZrO_3_	4

† *oS* is the base-centred orthorhombic lattice, *i.e.* either *A*, *B* or *C*.

‡ |*c*/*b* − 1| ≃ 0.005.

§ ≡ |**a**_1_ + **a**_2_|.

¶ |*a*/*b* − 1| < 0.001, |*c*/*b* − 1| < 0.004.

†† |*a*/*b* − 1| < 0.003, |*c*/*b* − 1| < 0.001, *i.e.* pseudo-cubic or pseudo-tetragonal.

**Table 4:** Structure amplitudes 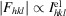 for element structure types If *h*, *k*, *l* do not satisfy any of the specified conditions, |*F*_*hkl*_| = 0.

Structure type	Lattice	|*F*_*hkl*_|	Conditions
*A*1, Cu (f.c.c.)	*F*	4(*f*)	*h*, *k*, *l* odd/even
*A*2, W (b.c.c.)	*I*	2(*f*)	*h* + *k* + *l* = 2*n*
*A*3, Mg (h.c.p.)	*P*	1(2*f*)	*h* + 2*k* = 3*n* and *l* even
		1(*f*)	*h* + 2*k* = 3*n* ± 1 and *l* even
		1( 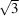 *f*)	*h* + 2*k* = 3*n* ± 1 and *l* odd
*A*4, diamond	*F*	4(2*f*)	*h* + *k* + *l* = 4*n*
		4( 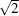 *f*)	*h*, *k*, *l* odd

**Table 5:** Structure amplitudes 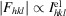 of some structure types of *AB* compounds If *h*, *k*, *l* do not satisfy any of the specified conditions, |*F*_*hkl*_| = 0.

Structure type	Lattice	|*F*_*hkl*_|	Conditions
*B*1, NaCl	*F*	4(*f*_*A*_ + *f*_*B*_)	*h*, *k*, *l* even
		4(*f*_*A*_ − *f*_*B*_)	*h*, *k*, *l* odd
*B*2, CsCl	*P*	*f*_*A*_ + *f*_*B*_	*h* + *k* + *l* = 2*n*
		*f*_*A*_ − *f*_*B*_	*h* + *k* + *l* = 2*n* + 1
*B*3, zincblende (ZnS)	*F*	4(*f*_*A*_ + *f*_*B*_)	*h*, *k*, *l* even and *h* + *k* + *l* = 4*n*
		4(*f*_*A*_ − *f*_*B*_)	*h*, *k*, *l* even and *h* + *k* + *l* = 4*n* + 2
		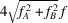	*h*, *k*, *l* odd

**Table 6:** Shortest translation vectors 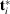 in reciprocal space for all lattice types; they represent the basis vectors of the respective primitive unit cell

Lattice	Lattice point density	Reciprocal translation vectors 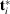
*P* 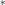	1	(1, 0, 0) 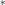 , (0, 1, 0) 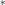 , (0, 0, 1) 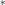
*A* 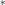	1/2	(1, 0, 0) 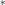 , (0, 1, 1) 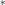 , 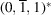
*B* 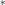	1/2	(0, 1, 0) 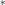 , (1, 0, 1) 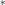 , 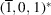
*C* 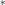	1/2	(0, 0, 1) 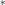 , (1, 1, 0) 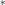 , 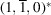
*I* 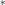	1/2	(1, 1, 0) 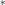 , (1, 0, 1) 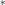 , (0, 1, 1) 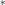
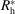	1/3	(1, 0, 1) 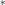 , 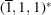 , 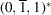
*F* 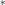	1/4	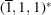 , 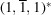 , 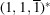

## Footnotes

1 A typical example is cubic sphalerite (ZnS) versus tetragonal chalcopyrite (CuFeS_2_) with *c*/*a* ≃ 2, but many others are described by Müller (2013 ▸).

2 Hexagonal phases are treated like trigonal ones, *i.e.* the fundamental sector is generated twice, independently and with different resolution.

3 The curve shape of *f*_el_ can be also derived from *f*_X_ using the Mott–Bethe formula: *f*_el_ = (*d* ^2^/10.4455)(*Z* − *f*_X_), including all surprising variations.

## Appendix A Reciprocal lattice of centred unit cells

The derivation of the lattice metric from a single BKD pattern is based on the correlative analysis of the reciprocal lattice (Li & Han, 2015 ▸; Nolze & Winkelmann, 2017 ▸). For primitive lattices this is comparatively straightforward. Unfortunately, many technically relevant phases are described by non-primitive Bravais lattices, such as ferrite and austenite, *i.e.* α-Fe and γ-Fe, respectively. Since the impact of unit-cell centring on the reciprocal lattice usually receives little attention, it will be briefly discussed here.

### A1. Cell transformation

To find the counterparts of centred cells in the reciprocal lattice, conversion to a primitive lattice description is recommended. Using the matrix _*X*_ **T**_*Y*_ as a description for a transformation from lattice type *Y* to *X*, the primitive descriptions of an *I* and an *F* lattice are, for example, given by the matrices

[see also *International Tables for Crystallography*, Vol. A, Table 3.1.3.1 (Aroyo, 2016 ▸) or Table 5.1.3.1 (Hahn, 2005 ▸)]. Their determinants |_*X*_ **T**_*Y*_| indicate the fraction of the volume of lattice *Y* compared with *X*. Therefore,  and  . For a reverse transformation the inverse matrices are valid, *i.e.*  ,

### A2. Proper indexing

_*X*_ **T**_*Y*_ and _*Y*_ **T**_*X*_ are very useful for correct indexing of lattice planes and directions. As pointed out by Nespolo (2017 ▸), only for primitive cell descriptions are *h*, *k*, *l* and *u*, *v*, *w* coprime integers. Thus, [*uvw*] may have a common factor of  or  for non-primitive cells, and in (*hkl*) the indices need no longer be prime but may also be a least-common multiple of 2 (*A*, *B*, *C*, *I*, *F*) or 3 (*hR*). Since it is not simple to see whether indices will be fractional or integer, it is easier to convert integer prime indices of the primitive lattice into those of the centred lattice using

Thus, it turns out that direction [100]_*P*_ in a primitive lattice description has the indexing of  in the centred *I*-lattice description, whereas [110]_*P*_ is also given by integers for *u*, *v* and *w* in the *I*-lattice description: [100]_*I*_. In contrast, lattice plane  transforms to (002)_*I*_, whereas the similarly indexed (111)_*P*_ changes to (222)_*I*_. Other lattice planes also keep the common description by prime integers after transformation: (001)_*P*_ changes to (110)_*I*_.

Since proper indexing refers to the next possible lattice point along a direction in the crystal or reciprocal lattice, for (*hkl*) and [*uvw*] the well known integral reflection conditions for *hkl* can be adapted [see *e.g.* Table 2.2.13.1 of Hahn (2005 ▸)]. For the just discussed *I* lattice, all indices in (*hkl*)_*I*_ must be doubled if *h* + *k* + *l* = 2*n* + 1, whereas for [*uvw*]_*I*_ all indices must be halved if *u* + *v* + *w* = 2*n*.

The matrix similarities  and  [equations (10) and (11)] already suggest some kind of relationship between the *I* and *F* lattices in direct and reciprocal space. Very often this is found in statements that the reciprocal lattice of *F* is an *I* lattice and vice versa. However, this is a rather phenomenological and misleading description. The fact is that a unit cell in reciprocal space derived from a centred lattice no longer has reciprocal-lattice points at each corner when their basis vectors are derived by

where *V*_uc_ is the volume of the unit cell. The resulting distributions of reciprocal-lattice points derived from centred cells and described by **a***, **b*** and **c*** are displayed in Fig. 10 ▸. As can be seen, only a subset of corners are occupied by (blue) lattice points.

For a first approximation it helps that the product of the numbers of lattice points in the real and reciprocal unit cells is always 1, regardless of the lattice type chosen. For example, since the *F* lattice contains four lattice points per centred unit cell, the derived reciprocal unit cell defined by (13) only contains ¼ lattice point; to show this graphically in Fig. 10 ▸, a volume of 2 × 2 × 2 cells needs to be considered (these eight cells contain  reciprocal-lattice points). From this it follows that **a***, **b*** and **c*** in centred reciprocal lattices are no longer translation vectors. Only the vectors  describe the translation symmetry of the reciprocal lattice. The shortest  are drawn as blue vectors in Fig. 10 ▸ and listed in Table 6 ▸, and they indicate **a***, **b*** and **c*** of the primitive cell.

Applying the translations  , a volume of 2 × 2 × 2 unit cells delivers for *F** a lattice point distribution which looks like an *I*-type lattice, whereas for *I** it appears like an *F*-type lattice [Figs. 3 ▸(*b*) and 3 ▸(*c*)].

For lattice types *A*, *B* and *C* their character remains, *e.g.* in the case of multiple cells for *C** the resulting reciprocal-lattice point arrangement also appears as a *C*-type lattice (Fig. 10 ▸).

For *R** the reciprocal basis vectors **a*** = **b*** and **c*** with α* = β* = 90° and γ* = 60° result. Of the resulting nodes in each layer of constant *l* **c*** in Fig. 10 ▸ only one-third are occupied by reciprocal-lattice points. This matches the expected lattice point density of  = 1/3 since  = 3. Note that for *l* = 1, 2 or 3 the distribution of points in each layer is shifted so that 3**c*** is the shortest translation vector along **c***, *i.e.* the reciprocal-lattice direction is correctly indexed by [003]*.
